# The Anorexigenic Effect of Serotonin Is Mediated by the Generation of NADPH Oxidase-Dependent ROS

**DOI:** 10.1371/journal.pone.0053142

**Published:** 2013-01-09

**Authors:** Xin-Ling Fang, Gang Shu, Jian-Jian Yu, Li-Na Wang, Jing Yang, Qing-Jie Zeng, Xiao Cheng, Zhi-Qi Zhang, Song-Bo Wang, Ping Gao, Xiao-Tong Zhu, Qian-Yun Xi, Yong-Liang Zhang, Qing-Yan Jiang

**Affiliations:** College of Animal Science, South China Agricultural University, Guangzhou 510642, China; Radboud University, The Netherlands

## Abstract

Serotonin (5-HT) is a central inhibitor of food intake in mammals. Thus far, the intracellular mechanisms for the effect of serotonin on appetite regulation remain unclear. It has been recently demonstrated that reactive oxygen species (ROS) in the hypothalamus are a crucial integrative target for the regulation of food intake. To investigate the role of ROS in the serotonin-induced anorexigenic effects, conscious mice were treated with 5-HT alone or combination with Trolox (a ROS scavenger) or Apocynin (an NADPH oxidase inhibitor) by acute intracerebroventricular injection. Both Trolox and Apocynin reversed the anorexigenic action of 5-HT and the 5-HT-induced hypothalamic ROS elevation. The mRNA and protein expression levels of pro-opiomelanocortin (POMC) were dramatically increased after ICV injection with 5-HT. The anorexigenic action of 5-HT was accompanied by markedly elevated hypothalamic MDA levels and GSH-Px activity, while the SOD activity was decreased. Moreover, 5-HT significantly increased the mRNA expression of UCP-2 but reduced the levels of UCP-3. Both Trolox and Apocynin could block the 5-HT-induced changes in UCP-2 and UCP-3 gene expression. Our study demonstrates for the first time that the anorexigenic effect of 5-HT is mediated by the generation of ROS in the hypothalamus through an NADPH oxidase-dependent pathway.

## Introduction

Serotonin (5-HT) is a classical neurotransmitter, which plays an important role in regulating food intake and energy homeostasis in mammals [1], [2], [3]. Administration of 5-HT or its analogue reduced food intake and stimulated energy consumption in both human and rats [4], [5]. 5-HT mediates the anorexigenic effect through its receptors in the hypothalamus, which modify the activities of neuropeptide Y/agouti- relative protein (NPY/AgRP) neurons and POMC neurons [6], [7], [8]. Because of the complexity and integrative properties of the central nervous system control of food intake, the intracellular mechanism by which 5-HT triggers the secretion of neuropeptides is poorly understood.

ROS, which are mainly produced by the electron transport chain [9] or NADPH oxidases [10], [11], [12], are important signaling molecules that induce oxidative stress and further contribute to DNA, protein and lipid damage [13]. Recently, emerging reports have confirmed a pivotal role of ROS in food intake and energy homeostasis regulation in the hypothalamus. For example, in response to an acute overload of nutrients, a sensitive elevation of the ROS concentration was sufficient to reduce food intake [14]. It has also been suggested that the central and peripheral administration of glucose can reduce food intake due to enhanced ROS levels in hypothalamus [15], [16]. Moreover, the gut-derived hormones, such as ghrelin and insulin, exert their central influences on energy homeostasis by controlling the hypothalamic ROS levels [17], [18].

In peripheral cells, it has been well established that 5-HT functions to elevate intracellular ROS levels [19], [20]. 5-HT can stimulate tyrosine phosphorylation and proliferation of smooth muscle cells through signaling pathways that utilize its transport system and O_2_^−^• formation [19]. Mukhina et al. also confirmed that transfection with the human 5-HT(1A) receptor induced the production of ROS in Chinese hamster ovary (CHO) cells. Additional signaling pathway analysis demonstrated that NADPH oxidase is located in the extracellular signal-regulated kinase (ERK) activation pathway, downstream of the G_i_βγ subunits and upstream of or at the level of the non-receptor tyrosine kinase Src. Therefore, the generation of both O_2_^−^• and H_2_O_2_ in CHO cells stimulated by the transfected 5-HT_1A_ receptor could account for the altered cellular redox properties through NADPH oxidase activity [20]. As expected, inhibitors of NADPH oxidase activity (diphenyleneiodonium and apocynin) dramatically blocked the elevation of ROS levels induced by 5-HT in peripheral cells [19], [20], [21], [22]. However, the effect of 5-HT on the generation of ROS in the hypothalamus remains unknown. It is not clear whether the anorexigenic effects of 5-HT are also mediated by the ROS.

To this end, we measured the food intake, hypothalamic ROS levels, free radical scavenging system enzyme activity, and NPY/POMC gene expression after ICV injection of 5-HT. The involvement of ROS and NADPH oxidase in the 5-HT-induced inhibition of food intake was evaluated by co-injection with a ROS scavenger (Trolox) or an NADPH oxidase inhibitor (apocynin). In this study, we demonstrate for the first time that the 5-HT-induced inhibition of food intake is mediated by the ROS signaling pathway. The results also confirmed the central role of NADPH oxidase in integrative appetite regulation, which may be important for the treatment of obesity and other metabolic diseases.

## Results

### 5-HT injection into the third ventricle decreased food intake

**Figure 1:**
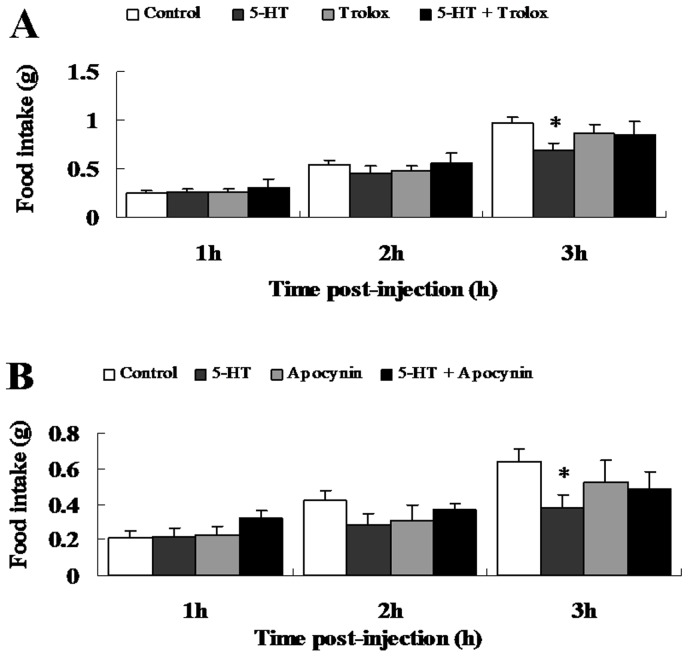
Effects of 5-HT, Trolox and Apocynin on food intake after third ventricle injection. Food intake was measured manually by weighing of pre-weighed food pellets at 1, 2 and 3 h post-injection. (A) ICV co-injection with 5-HT and Trolox. n = 11–12 per group. (B) ICV co-injection with 5-HT and Apocynin. n = 8–9 per group. Data are expressed as the means ± SEM. *, 0.01<p<0.05, vs. the control.

To study the role of hypothalamic 5-HT signaling in regulating food intake, we carried out three preliminary experiments (data not shown) to identify the optimal doses of 5-HT (2.5 nmol), Trolox (0.3 nmol) and Apocynin (0.9 nmol) [2], [18]. Compared with the vehicle-injected animals, 5-HT significantly decreased the accumulated food intake by 28.7% within 3 hours after injection, but not at 1 and 2 h (Figure 1). The significant interactions (p<0.05) showed the reversing effects of Trolox and Apocynin, which alone do not alter food intake, on the anorexigenic action of 5-HT (restored to 88.7% and 74.8% of Control, respectively, Figure 1A, B).

### NADPH oxidase–dependent hypothalamic ROS elevation is required for 5-HT-induced inhibition of food intake

**Figure 2:**
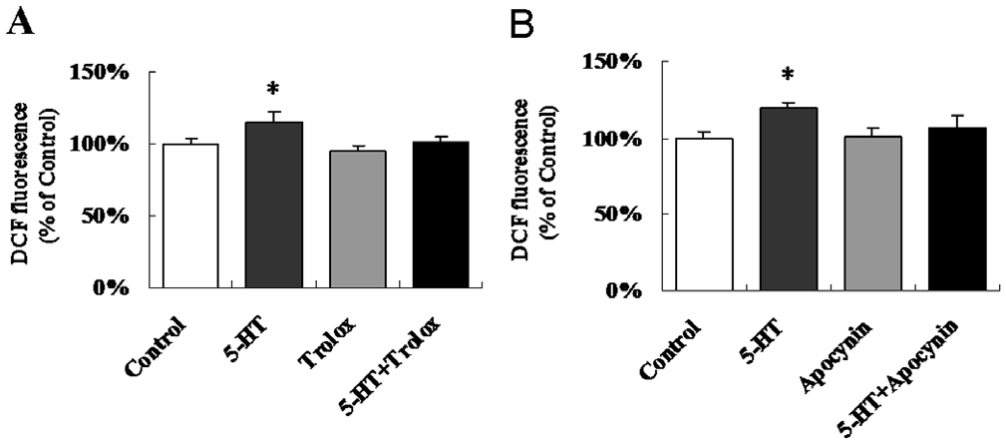
NADPH oxidase is required for the 5-HT-induced hypothalamic ROS elevation. The dye H_2_DCFDA was used to detect the change in the hypothalamic ROS levels. (A) ICV co-injection with 5-HT and Trolox. n = 11–12 per group. (B) ICV co-injection with 5-HT and Apocynin. n = 8–9 per group. Data are expressed as the means ± SEM. *, 0.01<p<0.05, vs. the control.

To determine whether 5-HT could induce ROS generation in the hypothalamus and whether NADPH oxidase was involved in hypothalamic ROS signaling to regulate food intake, we performed acute ICV co-injections with 5-HT and Trolox (a ROS scavenger) or Apocynin (a NADPH oxidase inhibitor). As expected, 5-HT could induce 19.1% and 15.6% ROS elevation, respectively. The significant interactions (p<0.05) showed that both Trolox (Figure 2A) and Apocynin (Figure 2B) completely prevented the 5-HT-induced ROS elevation without modifying the basal hypothalamic ROS levels.

### Effects of 5-HT on the activities of SOD and GSH-PX and on the MDA content in the hypothalamus

**Figure 3:**
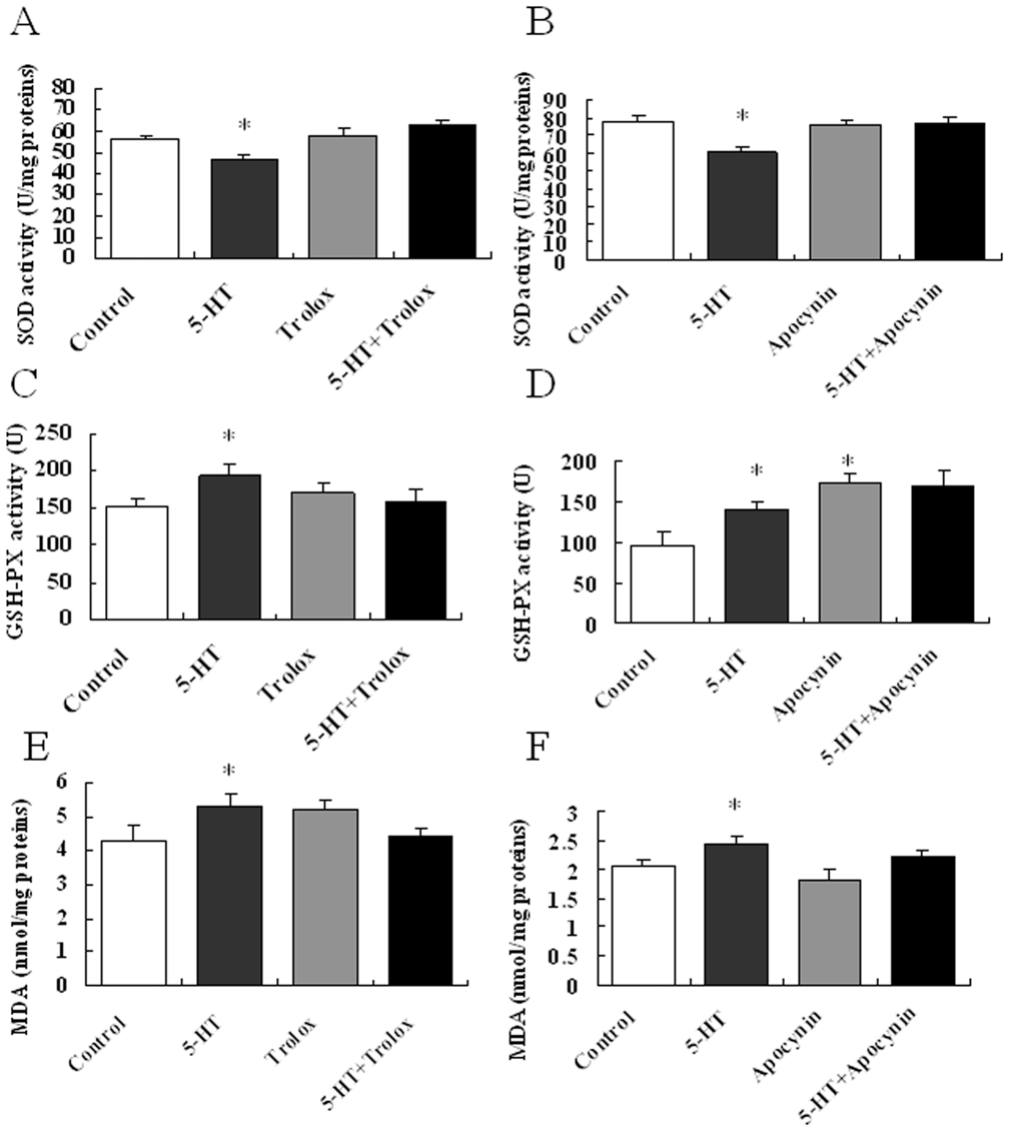
Effects of 5-HT on the activities of SOD and GSH-PX and on the MDA content in the hypothalamus. The total hypothalamic activities of SOD (T-SOD) and GSH-Px, as well as the malondialdehyde (MDA) levels, were determined using colorimetric assays. (A–B) Hypothalamic total SOD activity. n = 8–12 per group. (C–D) Hypothalamic GSH-PX activity. n = 8–12 per group. (E–F) Hypothalamic MDA content. n = 8–12 per group. Data are expressed as the means ± SEM. *, 0.01<p<0.05, vs. the control.

The accumulation of high levels of ROS induces oxidative stress and further results in protein and lipid damage [13]. To evaluate the stressful conditions in the brain antioxidant system that are induced by ICV injection of 5-HT, we determined the activity of the hypothalamic total SOD and GSH-PX as well as the MDA content. The T-SOD activity was inhibited, while the GSH-Px activity and MDA content were increased in the hypothalamus of 5-HT treated mice compared to the control group (Figure 3A–F). The activity of T-SOD and MDA content were statistically unaffected by Trolox or apocynin infusion. However, Apocynin, but not trolox, could increase the activity of GSH-PX (Figure 3D). Based on significant interactions (p<0.05), Trolox could reverse all of these effects, while Apocynin only reversed the decreased T-SOD activity induced by 5-HT (Figure 3A, B, C, E).

### ICV injection of 5-HT had opposing effects on the expression of UCP-2 and UCP-3 mRNA

**Figure 4:**
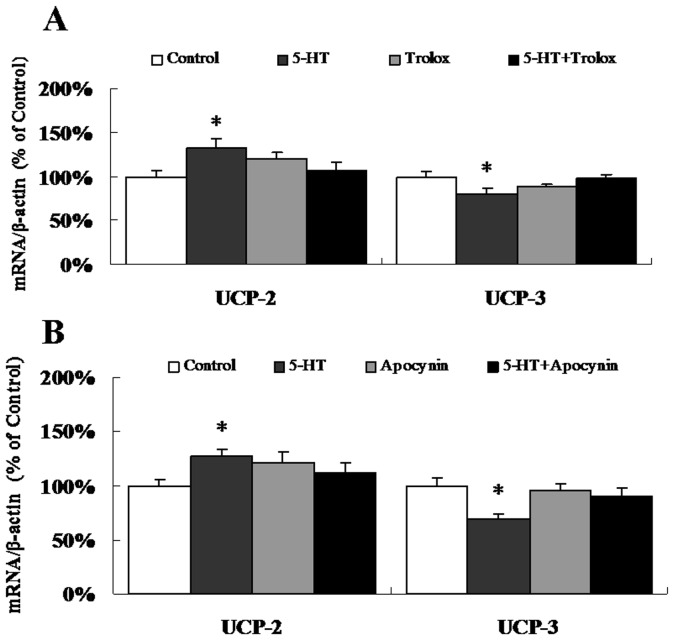
5-HT has contrasting effects on the expression of UCP-2 and UCP-3 mRNA. Real-time quantitative PCR was used to determine the mRNA levels of UCP-2 and UCP-3. (A) ICV co-injection with 5-HT and Trolox. n = 11–12 per group. (B) ICV co-injection with 5-HT and Apocynin. n = 8–9 per group. Data are expressed as the means ± SEM. *, 0.01<p<0.05, vs. the control.

We further evaluated the expression of UCP-2 and UCP-3 mRNA 3 h after 5-HT injection. The expression of UCP-2 mRNA was significantly increased, while the expression of UCP-3 mRNA was significantly decreased by the 5-HT ICV injection (Figure 4A, B). The significant interactions (p<0.05) showed that both Trolox and Apocynin could attenuate the effects of 5-HT on UCP-2 and UCP-3 mRNA expression (Figure 4A, B).

### 5-HT-induced NPY and POMC mRNA and protein expression

**Figure 5:**
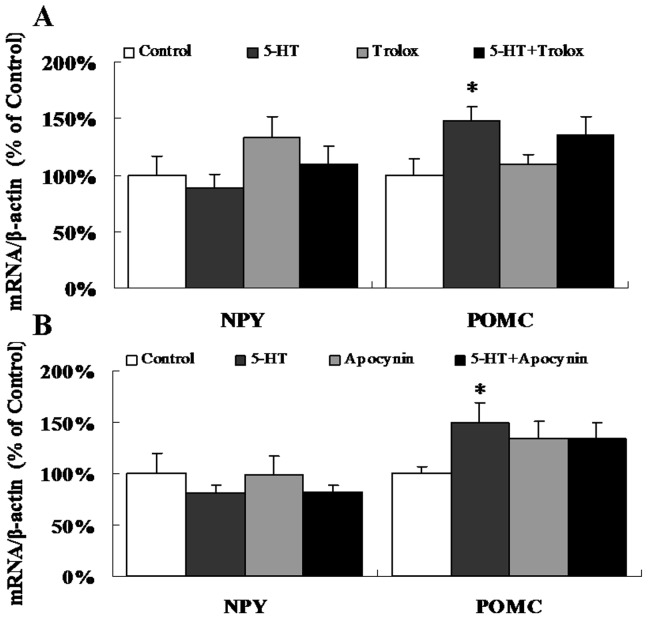
5-HT-induced NPY and POMC mRNA expression. Real-time quantitative PCR was used to examine the mRNA expression of NPY and POMC. (A) ICV co-injection with 5-HT and Trolox. n = 11–12 per group. (B) ICV co-injection with 5-HT and Apocynin. n = 8–9 per group. Data are expressed as the means ± SEM. *, 0.01<p<0.05, vs. the control.

**Figure 6:**
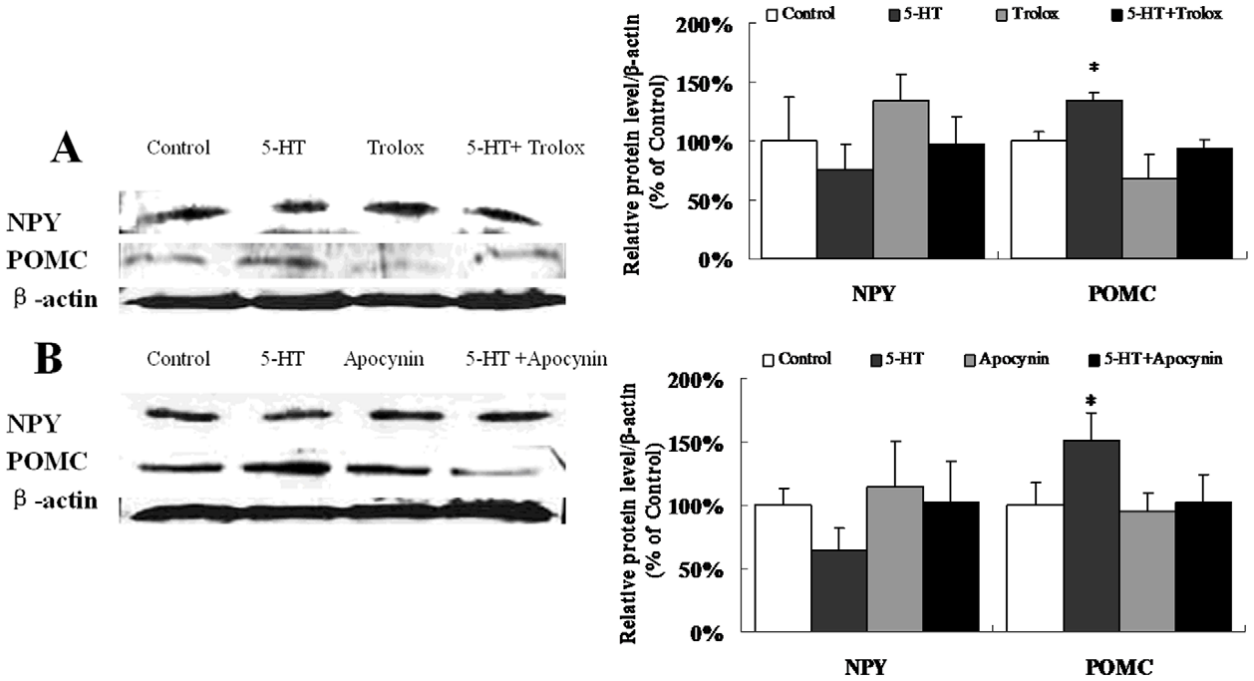
5-HT-induced NPY and POMC protein expression. Western blot analysis of NPY and POMC in the hypothalamus. (A) ICV co-injection with 5-HT and Trolox. n = 3 per group. (B) ICV co-injection with 5-HT and Apocynin. n = 3–4 per group. Data are expressed as the means ± SEM. *, 0.01<p<0.05, vs. the control.

After injection with 5-HT, there was a remarkable increase in the mRNA levels of POMC, while the mRNA levels of NPY were unchanged compared to the controls (Figure 5A, B). The protein levels of NPY and POMC induced by 5-HT were consistent with the mRNA levels (Figure 6A, B). However, the interaction analysis showed that both Trolox and Apocynin, which alone were unable to regulate POMC expression, could block the up-regulation of POMC caused by 5-HT on protein levels (p<0.05), but not on transcript levels (Figure 6A, B). This would suggest the post translational effect of Apocynin and Tolox on blockage of 5-HT-induced POMC expression.

## Discussion

The anorexigenic effect of 5-HT has been widely demonstrated in both mammals and birds. ICV or IP infusion of an 5-HT agonist decreased short-term (0.5 or 1, 2, 4 h) food intake only at higher doses, but not at lower doses, in rats previously deprived of food for 18 h [23], [24]. ICV injection of 5-HT in pigeons deprived of food for 24 h induced strong hypophagic effects 1 h after injection [25]. In our study, 5-HT injection significantly inhibited food intake in mice 3 h after injection, but not after 1 h or 2 h (Figure 1A, B). The differences between the previous study and our results may be due to the length of food deprivation, drugs used, concentration of the drugs or the method of injection.

5-HT fibers are in contact with the NPY/AgRP neurons and POMC neurons in the arcuate (ARC) and paraventricular nuclei (PVN), which are the pivotal areas that regulate energy balance [26]. We demonstrated that ICV 5-HT injection significantly inhibited food intake 3 h after injection (Figure 1A, B). This effect of 5-HT was also evident in terms of the mRNA and protein levels of POMC, which were significantly increased, although there were no significant differences in NPY levels compared to the control (Figure 5A, B; Figure 6A, B). The previous research has indicated that central 5-HT systems directly activate the POMC neurons [27]. ICV injection of 5-HT increases the mRNA expression of POMC [28]. However, administration of a 5-HT receptor agonist significantly reduced food intake and increased the mRNA expression of POMC, but there were no significant effects on the mRNA levels of NPY or AgRP [29]. Acute administration of fenfluramine, which can induce the release of 5-HT, did not alter NPY secretion in the PVN of food-deprived rats [30]. The different responses of NPY and POMC expression to 5-HT administration may be explained by divergent ROS metabolism strategies in different neurons [17], [31]. ROS generation is not merely a byproduct of substrate oxidation but instead has a crucial role in regulating neuronal responses in a substrate-dependent manner. For example, the NPY/AgRP neurons can activate a feed-forward buffering mechanism to scavenge ROS, but the POMC neurons do not seem to trigger such a buffering mechanism and fire at high levels when ROS accumulate in these cells [17]. Besides action potentials of neurons, POMC gene expression was also regulated by ROS generation. It was reported that increased ROS generation in the oxidative redox state attenuate the repression on POMC gene expression in pituitary corticotroph cells [32]. In addition, increased ROS production leads to the activation of ROS-related enzymes [33] and p38 MAPKs [34], [35], in which pathways expression of POMC was modulated to mediate appetite control. These evidences suggested that the POMC neurons might be more sensitive to the ROS generation. However, it needs to be investigated that whether 5-HT specifically increase the ROS generation in POMC neurons, but not NPY neurons.

Our data confirmed for the first time *in vivo* that 5-HT can potently induce an elevation of ROS levels in the central nervous system, consistent with the results observed upon intralipid injection, which provides essential fatty acids [14]. Andrews et al. reported that the ROS levels in POMC neurons of wild-type mice decreased while the ROS levels in NPY/AgRP neurons of UCP-2-knockout mice were elevated after IP injection with ghrelin [17]. Moreover, third ventricle injection of insulin induced the release of ROS in the hypothalamus, which resulted in the inhibition of food intake [18]. The results of the present study indicated that 5-HT-induced ROS elevation was also required for the inhibition of food intake (Figure 1A, Figure 2A). These data strongly support the hypothesis that the cellular ROS play a key role in the hypothalamic signaling pathways involved in the regulation of food intake and energy homeostasis, especially by controlling nutrient sensing [14] and hormonal signaling [17], [18].

Generally, ROS are produced from enzymatic and non-enzymatic sources. The enzymatic sources include NADPH oxidases, cytochrome P450-dependent oxygenases and xanthine dehydrogenase [10], [11], [12]. Several compelling lines of evidence suggest the presence of NADPH oxidase homologues in rodent brain tissue [36], [37], [38]. It has been demonstrated that 5-HT can modify the redox conditions in SMC and CHO fibroblasts in an NADPH oxidase-dependent manner [19], [20], [21], and 5-HT_2B_ receptors coupled to the NADPH oxidase-TNF-α converting enzyme (TACE) cascade are functionally coupled to ROS synthesis [21]. Our results also confirmed that NADPH oxidase-dependent ROS elevation was required for the central effect of 5-HT on food intake and hypothalamic ROS content, as well as for the altered POMC mRNA and protein expression. Although it had been established that the levels of ROS in the hypothalamus are induced by 5-HT, whether the intracellular mechanism of 5-HT functioned directly through the 5-HT receptor signaling pathway coupled to NADPH oxidase or indirectly through the increase in NAPDH generation was in need of further study.

The relationship between intracellular energy-sensing in hypothalamic neurons and the cascade of events that follows energy substrate oxidation involves uncoupling proteins (UCPs) [17], [39]. Negre-Salvayre et al. suggested a role for the UCPs in reducing mitochondrial ROS production [40]. UCPs are generally thought to be activated by ROS or ROS by-products to induce proton leak, thus providing a negative feedback loop for mitochondrial ROS production [41]. Our results showed that UCP-2 expression was increased, while UCP-3 expression was decreased, with the ROS elevation after ICV injection of 5-HT (Figure 4A, B). The opposite responses of UCP-2 and UCP-3 might be due to their distribution and activity in the hypothalamic neurons. UCP2 is abundantly expressed in the mouse brain [42] and is highly expressed in the hypothalamus, including in the NPY/AgRP neurons of the arcuate nucleus [43], [44]. On the other hand, UCP-3 is expressed predominantly in skeletal muscles and brown adipose tissue (BAT) [45], [46]. It has been demonstrated that the endogenous ROS-buffering mechanism in NPY/AgRP neurons is mediated by UCP-2, but not by UCP-3 [40]. However, the POMC neurons appear to be devoid of this UCP-2-dependent buffering mechanism, even though ROS accumulate in these cells [17]. Furthermore, it seems that only low, but not high levels of ROS are capable of activating UCP-3 [41], [47]. Although the UCP-3 expression in POMC neurons remains unclear, we speculate that the development of satiety might associated with the interaction between the accumulation of ROS and the reduction of UCP-3. Thus, the activity of different UCPs might be expected to be an important target in appetite regulation.

The accumulation of high levels of ROS induced oxidative stress [13], which involves the antioxidant defense systems. SOD is often regarded as the first line of defense against an upswing in ROS and is responsible for the conversion of superoxide to H_2_O_2_ in the cytoplasm and mitochondria [48]. Thus, 5-HT treatment induced a transient elevation of O_2_^-^•, which in turn is converted to H_2_O_2_ by SOD [49]. In the present study, the accumulated ROS in hypothalamus induced by 5-HT might also predominantly form H_2_O_2_. As a result, the T-SOD activity is inhibited by the excessive H_2_O_2_ (Figure 3A, B). Thus, GSH-Px, which had a major function in the detoxification of peroxides, will be activated to scavenge the excessive H_2_O_2_. Because the expression of GSH-Px in brain is very low [50], the results of trolox alone or co-treated with 5-HT suggests that the GSH-Px activity in hypothalamus might play an important role in scavenging excessive H_2_O_2_, which in turn regulates food intake. However, GSH-Px catalyses not only ROS, but also other organic hydroperoxides [51]. In addition, it has been reported that enhancing the rate of NADPH oxidation inhibits the activity of GSH-Px [52]. This may explain the increased GSH-Px activity after Apocynin injection (Figure 3D). Therefore, the increased GSH-Px activity may contribute to the destruction of other organic hydroperoxides and maintain the basal MDA level as showed in present results (Figure 3F), while the total ROS and food intake were unchanged.

In summary, our study demonstrates for the first time that the inhibitory effects of 5-HT on food intake are mediated by the generation of high ROS levels in the central nervous system through a NADPH oxidase-dependent pathway. Meanwhile, the free radical scavenging system appears to be feedback-activated.

## Materials and Methods

### Reagents and antibodies

5-HT, Trolox and Apocynin were purchased from Sigma-Aldrich (Sigma, St. Louis, MO, USA). The 2′,7^′^-dichlorofluorescein diacetate (H_2_DCFDA) was from Molecular Probes (Molecular Probes, USA). The NPY and POMC antibodies were from Phoenix Pharmaceuticals (Phoenix Pharmaceuticals, Belmont, CA). The other reagents were described throughout the text.

### Animal studies and ICV infusions

Seven-week-old male Kunming mice were purchased from the Guangdong Province Administration Office of Laboratory Animals. The Animal Ethics Committee of South China Agricultural University approved the guidelines for the care and use of animals (SCAU-AEC-2010-0416).

The animals were kept on a 12-h dark/light cycle, with the light off between 19:00 and 07:00. The animals had free access to food and water ad libitum. For the intracerebroventricular (ICV) infusion experiments, 15–20 g mice were anesthetized and then underwent stereotaxic surgery to implant a chronic stainless steel cannula (RWD Life Science, China) 10 d before the experiment. The placement coordinates for the third cerebral ventricle were the following coordinates from bregma: anterior-posterior, −0.8 mm; dorsal-ventral, −4.3 mm; and medial-lateral, 0 mm. The position of the cannula was verified at the end of the experiments by 0.5% Evans blue dye administration before animal was killed. Only those animals showing correct cannula position were included in data analysis. The mice were finally housed individually and were allowed one week for recovery. Before the experiments, the mice were handled daily and were adapted to intracerebroventricular injection by exposing them to at least two mock trials with saline, in which the injection needle was inserted without injection. The ICV injections were performed in awake mice. The injections consisted of either 2.5 nmol 5-HT (Sigma-Aldrich), 0.3 nmol Trolox (Sigma-Aldrich), 0.9 nmol Apocynin (Sigma-Aldrich), or vehicle. The 5-HT, Trolox and Apocynin were prepared as stock solutions in saline, 1% DMSO and 1% ethanol, respectively. Using a microsyringe pump (RWD Life Science, China) for injections (2 µL), all drugs and their respective vehicles were diluted in 0.85% NaCl. The injections were performed over 2 min. After injection, the cannula was kept in place for an additional 2 min to allow the drugs to diffuse away from the cannula tip. The animals were sacrificed by decapitation 3 h later, and the brains were immediately removed, and the hypothalami were excised according to the dissection described by Freitas et al [52]. Briefly, hypothalamus was isolated from the other brain regions by a cutting passing through the anterior part of the optic chiasm, behind the mammilary body, dorsally at the top of the third ventricle and laterally by a cutting bordering the optic tract.

### Food intake study

The food was removed 2 h before the ICV injection. After the ICV injection in the dark cycle, the mice were returned to their cages with food. The food intake was measured manually by weighing the pre-weighed food pellets at 1, 2 and 3 h post-injection.

### Hypothalamic ROS detection

The dye 2′,7^′^-dichlorofluorescein diacetate (H_2_DCFDA; Molecular Probes) was used to detect the change in hypothalamic ROS levels [18]. Briefly, after ICV injection, the hypothalami were dissected and immediately frozen in liquid nitrogen. After rapid thawing, the samples were homogenized with a Dounce homogenizer in ROS buffer. The homogenates were exposed to H_2_DCFDA and incubated in the dark at 37°C for 30 min. The reaction was stopped with ethanol and HCl. The homogenates were then centrifuged at 3,000 g for 15 min at 4°C. The supernatants were neutralized with NaHCO_3_ and centrifuged at 6,000 g for 15 min at 4°C. The ROS levels were determined by measuring the fluorescence strength with a BioTek Synergy 2 microplate reader (Winooski, VT, USA). The intensity of the fluorescence was expressed as arbitrary units per microgram of proteins.

### Hypothalamic T-SOD, GSH-PX and MDA assay

The hypothalamic homogenates were obtained by homogenizing frozen hypothalamus tissue (−80°C) in cold physiologic saline (4°C). The homogenates were then centrifuged at 3,000 g for 15 min at 4°C, and the protein concentrations of the supernatants were determined using the BCA Protein Assay Kit (BioTeke Corporation, China).

The total hypothalamic activities of SOD (T-SOD) and GSH-Px, as well as the malondialdehyde (MDA) levels, were determined using colorimetric assays with commercial kits (Nanjing Jiancheng Biotechnology Institute, China).

The hypothalamic T-SOD activity was measured using the hydroxylamine assay with a colorimetric method at 560 nm, and the results are presented as U per milligram of protein in the hypothalamus. The GSH-Px activity was determined by the GSH consumption speed, based on the reaction in which GSH is converted to GSSG at the same time as H_2_O_2_ is reduced to H_2_O. The MDA levels were determined using a spectrophotometer at 532 nm to monitor the reaction with 2-thiobarbituric acid (TBA). The results are presented as nmol per milligram of protein in hypothalamus.

### Real time RT-PCR

Total RNA (1 µg) was extracted from the hypothalamus using TRIZOL reagent (Invitrogen), converted to cDNA and used to determine the expression levels of NPY, POMC, UCP-2, UCP-3 and β-actin. The specific primers for Real-time quantitative PCR were as followed: 5′- TTGGGCATTCTGGCTGA -3′ (sense) and 5′- GATTGATGTAGTGTCGCAGAGC -3′ (antisense) for NPY, 5′- TTAAGCCGGTGGGCAAGA -3′ (sense) and 5′- GGACCTGCTCCAAGCCTAAT -3′ (antisense) for POMC, 5′- GTTGACCTCCCTTGCCACTT -3′ (sense) and 5′- CCCAAGCGGAGAAAGGAA -3′ (antisense) for UCP-2, 5′- ATGATACGCCTGGGAACTG -3′ (sense) and 5′- TCCAAGTCCCTTTCCACAG -3′ (antisense) for UCP-3 and 5′- CCACGAAACTACCTTCAACTC -3′ (sense) and 5′-TGATCTCCTTCTGCATCCTGT -3′ (antisense) for β-actin. Real-time quantitative PCR was performed using standard protocols on a Stratagene Mx3005P sequence detection system (Stratagene, USA). β-actin transcription was used as a reference, and the results are expressed as the ratio relative to β-actin expression.

### Western blotting

Mice were sacrificed by decapitation, and the brains were immediately removed. The hypothalami were dissected immediately and frozen in liquid nitrogen. The samples were lysed in radio immunoprecipitation assay (RIPA) buffer containing protease inhibitors. The total protein concentration was determined by BCA protein assay. After separation by 10% SDS-PAGE, the proteins were transferred to polyvinylidene difluoride (PVDF) membranes and blocked with 5% skim milk. The following antibodies were used: NPY and POMC (1∶500, Phoenix Pharmaceuticals), β-actin (1∶1000, Abcam). All primary antibody incubations were performed at 4°C overnight, followed by incubation with the secondary antibodies (1∶10000, Odyssey) for 1 h at room temperature. The signals were detected using an Infrared Imaging System (LI-COR CO., Lincoln, NE). The protein expression was normalized to β-actin.

### Statistical analysis

The values are expressed as the means ± SEM. The statistical significance of the differences between separate treatment groups and control group were calculated using Student's t test. Two-way analysis of variance (ANOVA) was used to test the interaction between 5-TH and Trolox or Apocynin (SPSS 17.0, Chicago, IL). The differences among groups were considered significant at P<0.05.
